# Management of refractory pruritus in Alagille syndrome with dupilumab treatment: A case report

**DOI:** 10.1016/j.jdcr.2024.06.030

**Published:** 2024-07-08

**Authors:** Daniel S. Alicea, Rachel Santana Felipes, Beth N. McLellan

**Affiliations:** Division of Dermatology, Department of Medicine, Albert Einstein College of Medicine, Montefiore Medical Center, Bronx, New York

**Keywords:** Alagille syndrome, dupilumab, genetic liver disease, maralixibat, multidisciplinary management, pruritus

## Introduction

Alagille syndrome (ALGS) is an autosomal dominant multisystem disorder with a reported incidence of 1:30,000 live births. ALGS-associated cholestasis presents in infancy and manifests with severe pruritus often leading to scratching, skin scarring, and resulting lichenoid processes. In fact, pruritus has been considered one of the most troublesome symptoms in this syndrome. Medical management for pruritus secondary to ALGS remains mostly targeted on supportive care with a variety of options including rifampin, ursodiol, and ileal bile acid transport inhibitors with promising results. However, in refractory cases, the use of biologics may be needed. Here, we present a case of a patient with chronic, refractory pruritus in the setting of ALGS successfully treated with dupilumab.

## Case report

**Fig 1:**
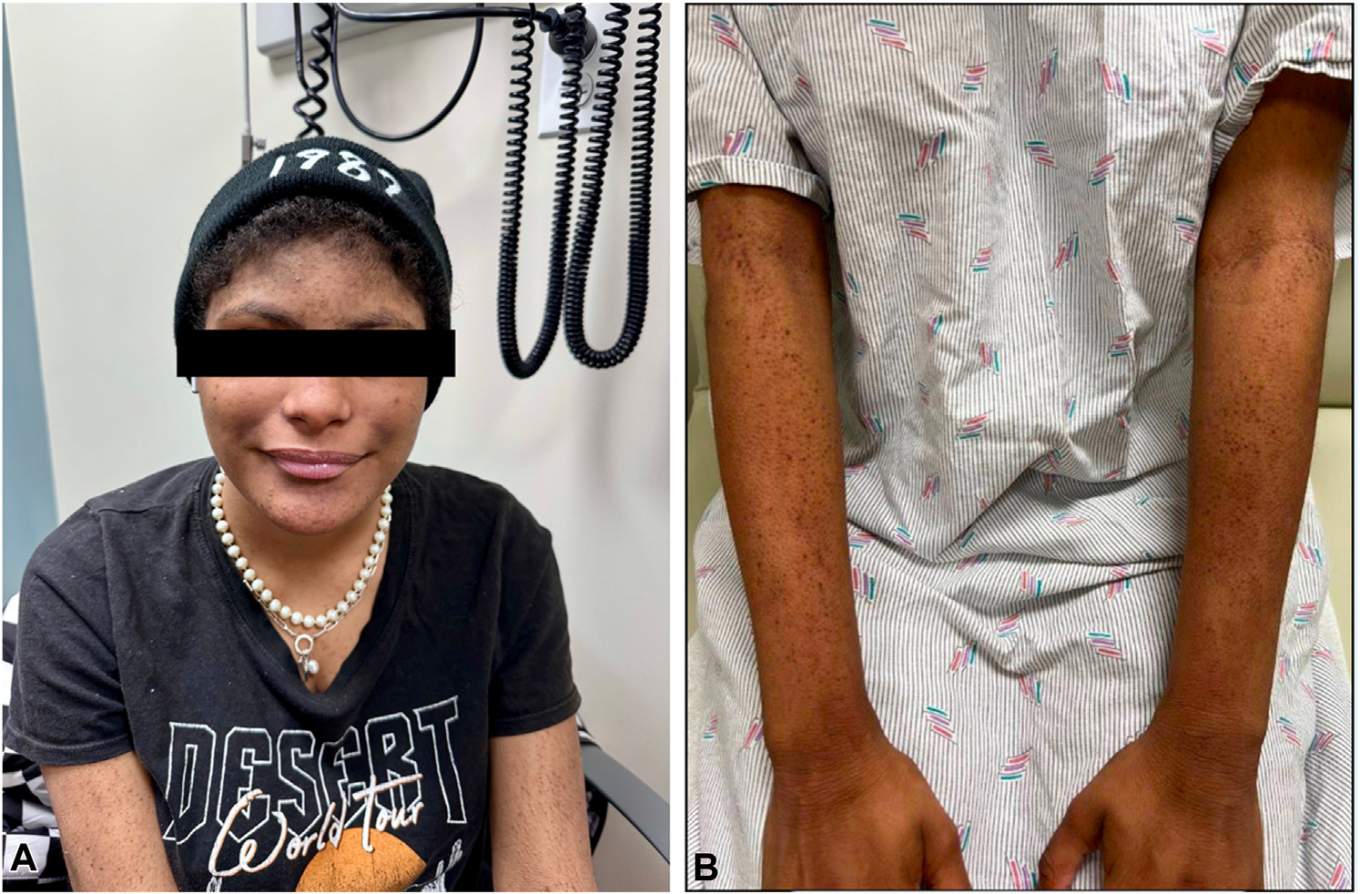
**A,** Patient presents with a prominent forehead and a straight nose; classical features of Alagille syndrome. **B,** Diffuse papules at initial presentation.

An 18-year-old woman with ALGS diagnosed in Puerto Rico, presented to dermatology for a lifelong itch, rated 9.5/10 in intensity, unresponsive to initial treatments with rifampin and ursodiol. Maralixibat chloride had been discontinued due to a facial rash. On physical examination, the patient displayed facial features consistent with ALGS, including a prominent forehead and a straight nose with a flattened tip (Fig 1, *A*). Additional examination showed diffuse papules on the extremities which were clinically consistent with lichen amyloidosis secondary to chronic pruritus (Fig 1, *B*). Due to the refractory and chronic pruritus, the patient was started on dupilumab 600 mg loading dose and then 300 mg every other week with triamcinolone 0.1% ointment twice a day. At 6-week follow-up, the patient noted significant improvement in itch, rating her pruritus a 6/10 in intensity, and will continue dupilumab 300 mg biweekly.

**Fig 2:**
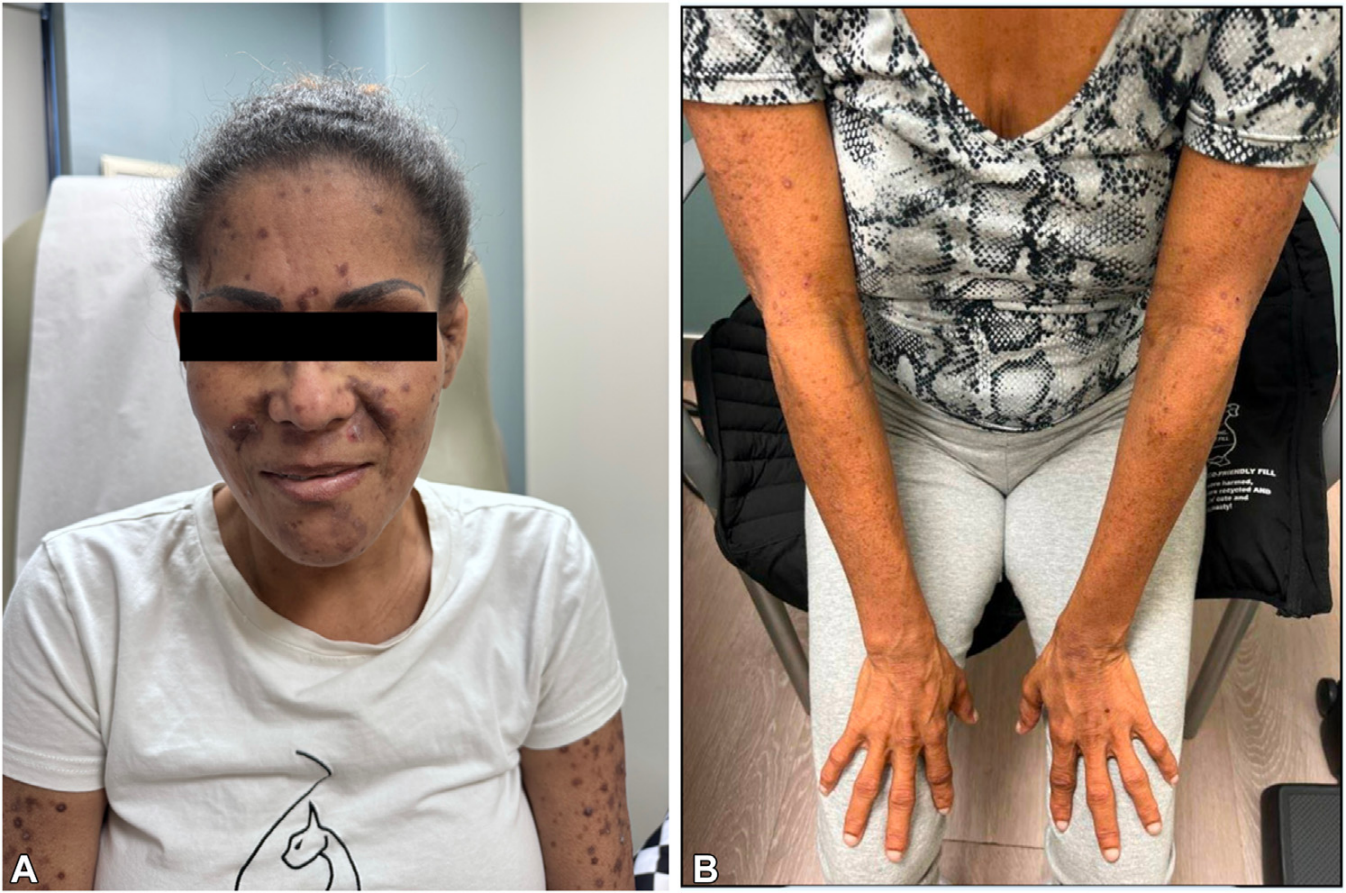
**A,** Facial features consistent with Alagille syndrome including a prominent forehead, bulbous tip of the nose, and pointed chin. **B,** Diffuse xerosis and firm papules noted on arms bilaterally at initial presentation.

The patient’s mother, a 45-year-old woman from Puerto Rico with metastatic hepatocellular carcinoma (HCC), initiated on treatment with atezolizumab/bevacizumab, and diabetes mellitus, also presented to dermatology for lifelong itching and rash. Family history was notable for liver cancer in her father and paternal uncle. As her daughter, clinical examination of this patient displayed features concerning for ALGS, including a prominent forehead, a bulbous nose tip, and a pointed chin (Fig 2, *A*). Additionally, skin examination showed diffuse papules on the arms and legs which were clinically consistent with lichen amyloidosis (Fig 2, *B*). Given the strong family history of liver cancer and confirmed diagnosis of ALGS in her daughter, it was thought likely that this patient also had ALGS, causing both her pruritus and HCC. The patient was managed with triamcinolone 0.1% ointment twice a day with no improvement in itch at 6-week follow-up. Given diffuse involvement, the patient was initiated on dupilumab 600 mg once, then 300 mg every other week. At the 9-week follow-up, the patient noted no improvement in itch and reported a new rash. Examination showed the same diffuse papules but also new violaceous scaly papules suggestive of a lichenoid process on the back, face, and abdomen. Dupilumab was continued for both pruritus and new onset of likely immune checkpoint inhibitor-related lichenoid eruption.

## Discussion

ALGS is a familial disorder with structural and functional abnormalities, including an interlobular bile duct deficiency associated with cholestasis.^1^ Globally, only 500 cases of ALGS have been reported including 9 cases of ALGS in Cuba and no reported cases from other Caribbean islands. ALGS is present in one out of 70,000 to 100,000 newborns, with no sex preference, autosomal-dominant transmission, and variable expression with 30% of patients showing parental-inherited mutations. It is caused by defects of the Notch signaling pathway, most commonly due to mutations in the Jagged I gene (ALGS type 1) and in a small proportion of cases (1% to 2%) in the NOTCH2-gene (ALGS type 2), giving rise to abnormal angiogenesis and ductopenia.^2^ Diagnosis is based on the combination of at least 3 of the following criteria which are all complex features seen in ALGS: chronic cholestasis; cardiovascular abnormalities; vertebral arch defects; ocular abnormalities; characteristic facial appearance; or family history of the syndrome.^3^ The etiology of pruritus associated with cholestasis is unclear but has been postulated to result from the accumulation of toxic bile acids, steroid-derived metabolites, histaminergic pathways, or neurogenic endogenous ligands that interact with central opiate receptors.^4^^,^^5^ Cutaneous manifestations are rare but reflect the underlying metabolic alterations, ranging from lichen amyloidosis as a consequence of scratching,^6^ photosensitivity/porphyria cutanea tarda-like blistering secondary to abnormalities in porphyrin metabolism,^1^ eruptive xanthomas from high cholesterol concentrations and xerosis and follicular hyperkeratosis from low serum levels of vitamin A and zinc.^7^ These skin findings suggest the diagnosis in the absence of known anamnestic and laboratory information.^8^

Pruritus is the most common presenting symptom, occurring very early, often increasing with age. A 1991 case reported a 22-year-old woman with ALGS who presented with lifelong unbearable pruritus and biopsy-confirmed lichen amyloidosis.

ALGS is a risk factor for the development of HCC. Annual follow-up with serum alpha-fetoprotein combined with an abdominal computed tomography scan is recommended to screen for HCC. Treatment options for pruritus remain limited with the current standard therapy consisting of bile acids binding resins, antihistamines, phenobarbital, rifampicin, and ursodeoxycholic, alone or in combination.^4^ However, maralixibat, an ileal bile acid transporter inhibitor, remains the first drug approved by the US Food and Drug Administration for cholestatic pruritus secondary to ALGS in patients age ≥3 months.^9^ Ultimately, this syndrome is curable with either a hepatic resection or liver transplantation.

## Conclusion

Here, we present 2 cases of a mother and daughter with pruritus secondary to ALGS. To our knowledge, these are the first reports which: (1) describe the occurrence of ALGS in a family of Puerto Rican descent and (2) utilize dupilumab for refractory pruritus secondary to ALGS with mixed results. Although the second patient did not note a reduction in pruritus, she had additional complicating factors including active malignancy, immunotherapy, and a new likely drug-induced lichenoid eruption. Given the intractable and chronic nature of the pruritus caused by ALGS, further study into the use of dupilumab should be considered.

## Conflicts of interest

None disclosed.
